# Risk Behaviors Related to Inter-personal Violence Among School and College-going Adolescents in South Delhi

**DOI:** 10.4103/0970-0218.40874

**Published:** 2008-04

**Authors:** Rahul Sharma, Vijay L Grover, Sanjay Chaturvedi

**Affiliations:** Department of Community Medicine, University College of Medical Sciences, Delhi; 1 Department of Community Medicine, VMMC & Safdarjung Hospital, New Delhi, India; 2 Department of Community Medicine, MIMS, Ambala, Haryana, India; 3 Department of Community Medicine, UCMS & GTB Hospital, Delhi, India

**Keywords:** Violence, adolescents, risk behavior

## Abstract

**Background:**

Injuries are a major cause of death and disability among the adolescents in the world.

**Objective:**

To study risk behavior related to interpersonal violence amongst school- and college-going adolescents in South Delhi and its epidemiological correlates.

**Study Design:**

Cross-sectional study.

**Setting:**

Three schools and two colleges in South Delhi.

**Participants:**

Five hundred and fifty adolescents aged 14-19 years.

**Statistical Analysis:**

Proportions, Chi-square test, multivariate logistic regression.

**Results:**

Among the study participants, 65 (11.8%) reported having carried a weapon in past 30 days. Seventy-four (13.5%) respondents had threatened or injured someone with a weapon in past 12 months. Almost one in every two boys (49.1%) reported being involved in a physical fight in past 12 months. Involvement in interpersonal violence was found to be significantly more amongst males than females. Adolescents who were working part time were more likely to be ‘at risk’ (67.5%) than those not working (48.5%). In logistic regression analysis, the significant correlates of interpersonal violence were male gender, lower age, number of close friends, having seen role models smoke/drink, and residing in resettlement colonies, slums or villages. The findings regarding violence-related behaviors among adolescents are remarkably similar to those in other countries.

Injuries are a major cause of death and disability among the adolescents in the world. These include unintentional injuries such as involvement in road traffic accidents, injuries resulting from violence towards self such as suicides, or from interpersonal violence such as involvement in physical fights. In a large school-based international study of adolescent students in five American and European countries, the findings regarding violence-related behaviors were found to be consistent and remarkably similar across countries.(1) It is a moot question whether the violent behaviors among adolescents would be similar in urban cities of developing countries too.

The present study covered six categories of important health risk behaviors among adolescents. This study was carried out with the purpose of finding out the prevalence of various health risk behaviors among the school- and college-going adolescents in one region of Delhi and whether this prevalence is comparable with the international figures. It also sought to study the association, if any, of the health risk behaviors with various socio-demographic characteristics of the subjects. In this paper, the findings related to interpersonal violent behaviors among the school- and college-going adolescents are presented.

## Materials and Methods

The study was a cross-sectional analysis of the subject population. The units of the study were 14- to 19-year-old adolescents studying in various schools and colleges in South Delhi. The study was reviewed and approved by the review board in the parent institution UCMS, Delhi, before being carried out. The metropolitan city of Delhi is also the capital of India. It has a population of nearly 13.8 million (as per the 2001 Census of India), in an area of 1483 square kilometers. Since January 1997, the city has been divided into nine districts.(2) For the purpose of the present study, two districts - South and Southwest districts - were together considered the South Delhi region. All the schools and colleges in the region were included in the sampling frame. A two-stage cluster sampling design was used to draw a representative sample of students in classes 9-12^th^ in schools, and first two years of graduation in colleges. These classes were chosen as they correspond to the desired age group of 14- to 19-year-old adolescents.

The first stage was random selection of three schools from the list of schools using a table of random numbers. Similarly, two colleges were randomly selected from the list of colleges. The second stage comprised random sampling of one class each from standards 9-12^th^ in each selected school. In the selected colleges, two classes each were randomly chosen from the first and second years of graduation. All students in the selected classes, present on the day of the survey, were eligible to participate, allowing for anonymous and voluntary participation. At the time of data analysis, respondents who had stated their age to be either less than 14 years or more than 19 years were excluded. Written permission and consent from the principals was obtained prior to conducting the study in their schools/colleges. Written consent was also obtained directly from the subjects who were majors.

A pre-tested, semi-open-ended and self-administered questionnaire was used in the study. The information thus collected on the tools was converted into a computer-based spreadsheet. Among the respondents, the adolescents were identified as being ‘at risk’, if he or she answered in the affirmative to any of the individual risk behaviors comprising the particular domain. The association of risk behaviors with a range of socio-demographic factors of the adolescents was explored as part of the study. Cross-tabulations were analyzed and the statistical significance of differences between ‘at risk’ and ‘not at risk’ groups was checked by applying Chi-square test. Binary logistic regression was applied to analyze the relationship between risk behaviors and various independent variables under study.

## Results

The mean age of the respondents was 16.5 ± 1.5 years. Overall, among the 550 respondents, 67.1% were males and 32.9% were females. A large majority of respondents were Hindus (492, 89.6%). A majority of subjects (343, 62.4%) reported their place of residence as being a private colony or a separate bungalow. Three-fourths (407) belonged to nuclear families, and remaining were from joint families.

The prevalence of various facets of risk behaviors concerning interpersonal violence is shown in Table 1. The adolescents were asked about having carried a weapon such as knife, gun, stick, club, hunter, sword, etc., at any time during the 30 days prior to study. Among the study participants, 65 (11.8%) reported having carried a weapon in past 30 days. One reason for this number could be the fact that boys belonging to the Sikh religion are expected to carry a small knife (kirpan) with them for religious reasons. However, only 11 being Sikh boys (2%) among the 550 respondents, this reason would account for only a small fragment of the affirmative responses to the question.

**Table 1:** Prevalence of risk behaviors related with inter-personal violence among the respondents

Risk behavior	Males (*n* = 369) *n* (%)	Females (*n* = 181) *n* (%)	Total (*n* = 550) *n* (%)
Carried a weapon in past 30 days	58 (15.7)	7 (3.9)	65 (11.8)
Threatened or injured someone with a weapon any time in past 12 months	64 (17.3)	10 (5.5)	74 (13.5)
Involved in physical fight any time in past 12 months	181 (49.1)	37 (20.4)	218 (39.6)
Injured in physical fight any time in past 12 months	84 (22.8)	14 (7.7)	98 (17.8)
Involved in physical fight in school/college any time in past 12 months	142 (38.5)	11 (6.1)	153 (27.8)

A higher proportion of males (15.7%) than females (3.9%) reported having carried a weapon (*P* < 0.001). Seventy-four (13.5%) had threatened or injured someone with a weapon in past 12 months, a majority of this being males. Almost one in every two boys reported being involved in a physical fight in past 12 months. One in every five (20.4%) girls, with overall 39.6%, reported having being involved in a physical fight. Ninety-eight respondents (17.8%) reported having received an injury in a physical fight, which was serious enough to require medical attention, during the 12 months prior to study.

For studying associations, an adolescent who responded ‘yes’ to any of the five individual questions was considered ‘at risk’. As shown in Table 2, ‘at risk’ behavior was found to be more among respondents who were currently studying in or had done their schooling from government or government-aided schools (56.1%). Adolescents who were working for money besides studying were more likely to be ‘at risk’ (67.5%) than those not working (48.5%). A significant relationship was observed with age (*P* = 0.007) and gender (*P* ≤ 0.001) of the subject. The prevalence of inter-personal violence behaviors declined with increasing age-group. Males were more likely to be ‘at risk’ regarding these behaviors than females. A significant association was observed with the number of ‘role models’ that the adolescents had ever seen smoking or drinking (out of six asked about, namely father, mother, best friend, sibling, teacher and favorite celebrity). Prevalence of the risk behaviors was highest among the adolescents who had seen ≥3 role models smoke or drink (81.8%); it was 48.4% among those who had seen 1-2 models, and the least among those who had not seen any of their role models ever smoke or drink (38.2%).

**Table 2:** Association of risk behavior with interpersonal violence related to various socio-demographic variables

Study characteristic (number of respondents)	Number of respondents having risk behavior (%)	*P*-value
Age group in years		
14-15(155)	93 (60.0)	0.007
16-17 (238)	115 (48.3)	
18-19 (157)	67 (42.7)	
Total (550)	275 (50.0)	
Gender		
Male (369)	226 (61.2)	<0.001
Female (181)	49 (27.1)	
Total (550)	275 (50.0)	
School attended		
Government or government-aided (316)	177 (56.0)	<0.001
Private (234)	98 (41.9)	
Total (550)	275 (50.0)	
Type of residence		
Government colony (127)	49 (38.6)	0.003
Private colony/bungalow (343)	174 (50.7)	
Urban slum (62)	40 (64.5)	
Total (532)	263(49.4)	
Respondent working for income		
Yes (40)	27 (67.5)	0.022
No (507)	247 (48.7)	
Total (547)	274 (50.1)	
Number of close friends		
None (30)	11 (36.7)	0.007
1-3 (275)	124 (45.1)	
4 or more (241)	138 (57.3)	
Total (546)	273 (50.0)	
Role models seen smoking or drinking (out of six asked about)		
None (131)	50 (38.2)	<0.001
One or two (353)	171 (48.4)	
Three or more (66)	54 (81.8)	
Total (550)	275 (50.0)	

The total for some of the study characteristics is less than 550 due to missing responses

In the binary logistic regression analysis [Table 3], the significant correlates of interpersonal violence were found to be male gender [odds ratio (OR) 3.69, 95% confidence interval (CI): 2.36-5.77], having four or more close friends (OR 3.17, 95% CI: 1.29-7.71), having seen role models smoke/drink, and residing in resettlement colonies, slums or villages. Increasing age was associated with lesser involvement in violence (OR 0.51, 95% CI: 0.3-0.89).

**Table 3:** Binary logistic regression analysis showing significant correlates of risk behavior related with interpersonal violence

Correlates	Categories	Adjusted odds ratio (95% CI)	*P*-value
Gender	Female	1 (Reference)	-
	Male	3.69 (2.36-5.77)	<0.001
Age	14-15 years	1 (Reference)	-
	16-17 years	0.64 (0.39-1.05)	0.077
	18-19 years	0.51 (0.3-0.89)	0.017
Close friends	None	1 (Reference)	-
	1-3	1.74 (0.72-4.23)	0.219
	4 or more	3.16 (1.29-7.71)	0.012
Role models seen smoking or drinking	None	1 (Reference)	-
	1-2	1.86 (1.15-2.99)	0.011
	3 or more	6.5 (2.92-14.45)	<0.001
Type of residence	Government colony	1 (Reference)	-
	Private colony/bungalow	1.29 (0.79-2.11)	0.307
	Urban slum	2.73 (1.29-5.76)	0.008

## Discussion

The present findings are similar to those of Kishore *et al.* who found that 12.5% of urban male adolescents in South Delhi region had carried a weapon in the past 30 days.(3) Our findings agree with those of Smith-Khuri *et al.* who found that an average 10.7% students aged 11-16 years in five countries reported having carried a weapon in past 30 days.(1) Across the globe, about one-third to one-half of the adolescent students report involvement in physical fights,(4–6) and the present findings fit the trend.

‘At risk’ behavior was found to be more among respondents who were currently studying in or had done their schooling from government or government-aided schools. The students in government schools are more likely to be belonging to families with lower socio-economic backgrounds, and there may be more stressors and factors leading to aggressive manifestations in their environment. An interesting finding was that as the number of close friends reported by the subjects went up, so did the involvement in interpersonal violence (*P* = 0.007). Having a bigger social network and interaction with a larger number of peers can increase the chances of small disagreements or points of dispute aggravating into fights. A significant association of risk behavior related to interpersonal violence was observed with the number of ‘role models’ that the adolescents had ever seen smoking or drinking. The relationship between having seen important people in their lives smoke or drink and indulging in violence would be difficult to justify as being direct or causal. However, seeing one or more of role models in their environment resorting to any one risk behavior - smoking or drinking - may develop a feeling of acceptance of negative risk behaviors in their social circle. This may remove their inhibitions and lead them to adopt not only that but other health risk behaviors as well including interpersonal violence.

Interpersonal violence found more common in the lower ages corresponds with findings of earlier international studies.(1,7) A possible explanation is that with age, adolescents become more mature and gain skills to solve conflicts in nonviolent ways. Another possible reason hypothesized is that students involved in violence are more likely to drop out or to be expelled. Previous studies and reviews have noted that boys worldwide have higher rates of involvement in interpersonal violence and greater morbidity and mortality from the resulting injuries.(7–9) The findings in the current study match the international experience so far, and bolster the theory that males are more likely to be involved in interpersonal violence than female adolescents. This can be attributed to the psychological framework of the young male minds and an instinct to resort to aggression to resolve any matters, whereas rarely differences are found among females manifesting as overt physical violence.

A limitation of the present study is that the study was cross-sectional in nature and, therefore, it was not possible to ascribe causality to the associations found significant. Moreover, the findings and their interpretations are restricted to school- and college-going adolescents only. Further studies are needed to cover the groups of adolescents who are out of school or college, as the prevalence of health risk behaviors is likely to be higher among such adolescents. Moreover, qualitative research methods like focused group discussions can be utilized in further studies to have in-depth analysis of the reasons for violent behaviors amongst school- and college-going adolescents.

## Conclusion

A large school-based international study had found that the findings regarding violence-related behaviors were consistent and remarkably similar across geographically and culturally dissimilar developed countries.(1) As discussed, many of the findings of the present study closely match the prevalence observed in various countries. It could probably imply that adolescents across different nations are basically alike in their nature and in susceptibility and proneness to risk behaviors concerned with interpersonal violence, and the adolescents in India are no exception.
